# COVID-19 may lower quality of life when infections and deaths increase: A longitudinal study in the Peruvian jungle

**DOI:** 10.3389/fpsyt.2023.905377

**Published:** 2023-03-28

**Authors:** Jeel Moya-Salazar, Chris A. Villareal, Betsy Cañari, Belén Moya-Salazar, Karina Chicoma-Flores, Hans Contreras-Pulache

**Affiliations:** ^1^South America Center for Education and Research in Public Health, Universidad Norbert Wiener, Lima, Peru; ^2^Hospital Nacional Docente Madre Niño San Bartolomé, Lima, Peru; ^3^Hospital II-1 Moyobamba, San Martin, Peru; ^4^Faculties of Health Science, School of Medicine, Universidad Norbert Wiener, Lima, Peru; ^5^Infectious Unit, Nesh Hubbs, Lima, Peru

**Keywords:** COVID-19, quality of life (QoL), rural population, SARS-CoV-2, jungle, mental problems, Peru

## Abstract

**Background:**

Quality of life (QoL) is a multifactorial concept on the perception of the individual’s wellbeing underpinned by environmental, psychological, and physical factors. Several studies have shown changes in QoL in the COVID-19 pandemic and may be due to increases in mortality rates, however, no study has investigated this among Peruvian jungle dwellers. Here, we have sought to estimate the QoL of individuals before and after the increase in cases and deaths from COVID-19.

**Methods:**

A questionnaire-based longitudinal study was conducted in 102 inhabitants (mean 40.75 ± 7.49 years). The Spanish version of the WHOQOL-BREF was used in two stages: April and June. The first stage was accomplished before the first confirmed case of COVID-19, and the second stage was when the daily mortality rate was 3.5% with an incidence of 87%.

**Results:**

Sixty (54.8%) participants were women, 67 (61.9%) were >31 years, and 38 (34.5%) and 32 (29.1%) participants had primary and secondary education, respectively. In the first and second stage we obtained an overall mean QoL of 46.65 ± 23.2 and 35 ± 27.7 points, respectively. Individuals had significantly lower QoL in the face of increased deaths in physical (*p* = 0.001), mental (*p* = 0.028) and environmental (*p* = 0.001) health domains, with the latter having the greatest impact (51.84 ± 5.81 vs. 16.66 ± 5.55 points).

**Conclusion:**

Quality of life of Peruvian jungle dwellers is reduced during periods of increased mortality and incidence by COVID-19. Preventive strategies aimed at reducing the impact of COVID-19 on the mental health and global wellbeing of individuals living in the Amazon are recommended to Peruvian authorities.

## 1. Introduction

In an unprecedented event, the COVID-19 pandemic has escalated into a global health emergency affecting all countries, while spreading infections and deaths. Latin America has been the third region hardest hit by SARS-CoV-2 during 2020, due to the social, economic, and political context in which measures have been taken to reduce its spread with poorly organized health responses and a lack of social responsibility (1). Peru, a country of about 33 million people with significant demographic and cultural differences, has the longest restrictions since 15 March 2020, but has no decisive and real impact on COVID-19 infections and deaths (2). The lack of social response to abide by the rules, as well as an inefficient health response to care for and track suspected and positive cases, have triggered the population to be threatened by the physical, psychological and social effects of SARS-CoV-2 (3, 4).

Human wellbeing has also been affected by the pandemic. Several studies have indicated that COVID-19 can worsen the quality of life (QoL) of affected communities and that this depression can be more severe in populations with marked inequities and social and economic inequalities (5–7). In addition to the QoL disruptions associated with increased COVID-19 infections and deaths that lead to fear, loss of control, and confusion (8), the effectiveness of measures to control the spread of COVID-19 can also play a key role in community welfare. In fact, policies and interventions for many diseases tend to ignore the preferences and traditions of indigenous communities (9), which also affects their QoL.

The pandemic has impacted rural communities, as shown by the study by Mhossen et al. in Egypt, where mental health was the most affected dimension, and the study by Tulegenova et al. in Kazakhstan, where a third of the population has reduced their QoL during the pandemic (10, 11). In addition, QoL is poor in rural compared to urban populations during the pandemic (12) due in part to a lack of access to quality health care and in part to ethnic inequalities and health inequities that have increased mortality in the rural population at the beginning of the pandemic (13, 14).

The Amazon is one of the most important and widely affected regions by SARS-CoV-2 in the Americas and the Caribbean (15, 16). The factors that have favored the spread of SARS-CoV-2 in the Amazon are health [such as the lack of hospitals, water/drainage, or the pre-existence of tropical diseases such as dengue or malaria (17)], economic (farming population or merchant), and social (lack of internet, use of personal protective equipment, and limited land access) (18–20). Under these conditions, the activities to control and prevent COVID-19 have not had a real effect, causing infections and deaths, and altering the wellbeing of the jungle communities. The quality of life of the inhabitants of the Peruvian jungle has been affected even more by a lack of adaptation to the pandemic, as well as resources to prevent the spread of disease, illiteracy and poverty (20). The inhabitants of the Peruvian jungle with high rates of illiteracy and poverty have suffered a greater impact on their QoL due to the lack of adaptation to the pandemic, and resources to prevent the spread of the disease (20).

Although the Peruvian government is monitoring cases of COVID-19 among indigenous populations (21), once infections start to rise and deaths occur daily, there is a gap in understanding how COVID-19 affects QoL. Research on QoL during the pandemic has given an idea of how it worsens (13, 22, 23), but its correlation with the number of infected or dead has not been established, mainly in indigenous or rural communities where there has not been as much health care available (24). It is imperative to understand how the QoL of the indigenous communities of the jungle is affected to propose care policies during the lockdown by COVID-19 in Peru, since understanding the magnitude of wellbeing can generate equitable and fair national care.

We aimed to estimate the QoL of individuals from the Peruvian jungle during the first outbreak of COVID-19. Furthermore, in this study, we explore changes in dimensions of QoL before and after the increase of cases and deaths in the municipality of Moyobamba, Northerm Peru.

## 2. Materials and methods

### 2.1. Study design and participants

We designed a longitudinal-cohort study in Moyobamba, San Martin region, from April to June 2020. A total of 102 participants of 62 families took part in this study. Previously we informed them about the objectives and signing the informed consent. The individuals evaluated were categorized as middle class according to FONCODES (25). People living for the last 5 years in the surveyed household who were older than 18 years old and of both sexes are considered inclusion criteria. The questionnaire was applied through door-to-door scheduled visits that included three healthcare providers along Moyobamba. The interview with the members of each family was conducted by health personnel who use to carry out home visits within the regional COVID-19 epidemiological control team following national regulations (26). These safety regulations include the use of PPE (i.e., masks, face coverings), social distancing, and limiting movement according to permitted schedules. To prevent infection the virtual survey was shared on GoogleForms (Google, CA, USA) with an access link *via* WhatsApp or Instagram (Facebook, WA, USA) while the visit was taking place. Internet access was secured with the use of additional data provided by the research team. An access link to informed consent was also provided, and the average time to complete the survey was 9 min per person.

### 2.2. Instruments and study phases

**FIGURE 1:**
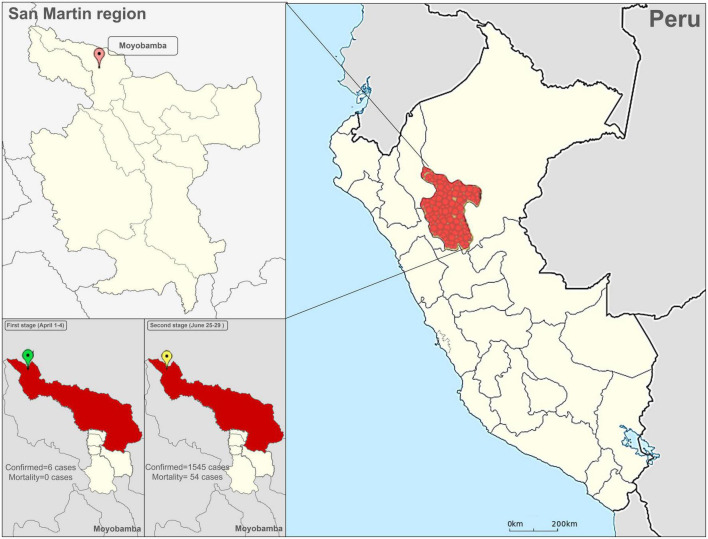
Location of the San Martin region in the Peruvian jungle. The confirmed cases and deaths from COVID-19 in the two stages of the study are shown in the lower tables.

To assess the QoL of quarantined families we used the self-administered World Health Organization Quality of Life Brief (WHOQOL-BREF) questionnaire consisting of 26 questions (five-item Likert-type scale) divided into four dimensions: physical, psychological, social relations, and environment (27). The higher score indicates better QOL among participants. The Spanish version of WHOQOL-BREF is an official version of WHO that has been made in a Spanish sample and that has had adequate validity (α > 0.88). The Spanish version of the WHOQOL-BREF was used for our study in two stages: the first stage was between April 1–4, and the second stage took place between June 25–29 (Figure 1). The first stage was accomplished before the first confirmed case of dead by COVID-19 in the San Martin region, and the second stage when the daily mortality rate was 3.5% with an incidence of 87% (1,545 confirmed cases) (28). In addition, we collected demographic data on age, gender, education level, drug use and previous disease, occupation, and contagion data (relative previously infected or died of COVID-19).

### 2.3. Statistical analysis

We use descriptive statistics for continuous variables (mean and standard deviation) and categorical (frequencies) according to demographic variables. The QoL was interpreted by following the WHOQOL-BREF (27). A Chi-square test was used to compare two categorical variables. To evaluate the differences in QoL scores in the first and second stages we used paired-samples *t*-test. In addition, one-way ANOVA with Bonferroni *post-hoc* test was used to determine the differences between the variables with more than two categories (i.e., educational level). We use a significance threshold of *p*-value < 0.05 and a 95% confidence interval. Statistical analysis was performed in IBM SPSS V22.0 (Armonk, NY, USA).

### 2.4. Ethical aspects

To comply with the ethical research guidelines, they first coordinated with the health managers of each municipality and with the local agents of each neighborhood to be able to report on the study days before it began. This study has been approved by the Norbert Wiener University Institutional Review Board (document FCE-RRR-COVID-2020.03-01) and follows the statement from Helsinki (29). In addition, we have used informed consent in each interview in both phases of the study and have provided communication with the participants after the study to inform them of the results and provide them with health care.

## 3. Results

### 3.1. Characteristics of populations

Participants had a mean age of 40.75 ± 7.49 years (95% CI 37.2 to 43.8) where 67 (61.9%) participants were older than 31 years, and 60 (54.8%) were women. Eighty-two (74.2%) participants were working and 67 (81.7%) had casual jobs. 12.7% of the participants suffered from previous chronic disease, a total of 20 (18%) participants had habitual drug use for the last 6 months (Table 1). Also, a total of 38 (34.5%) and 32 (29.1%) participants had primary and secondary education, respectively, being the most frequent answer.

**TABLE 1:** Demographic data of the participants (*N* = 110).

Variables	Categories	Frequency (*n*)	Percentage (%)
**Age (year)**			
	18–30	43	38.7
	31–50	32	29.2
	>50	35	31.8
**Gender**			
	Male	50	45.2
	Female	60	54.8
**Work**			
	Yes	82	74.2
	No	28	25.8
**Type of job (*n* = 82)**			
	Formal	43	52.4
	Informal	67	81.7
**Previous illness**			
	Yes	14	12.7
	No	96	87.3
**Drug consumption**			
	Yes	20	18.2
	No	90	81.8
**Educational level**			
	Illiterate	9	8.2
	Elementary	38	34.5
	High-School	32	29.1
	University/Technical	31	28.2

### 3.2. QoL during COVID-19

Regarding the questions about general wellbeing, the average was moderate, both in QoL and physical condition (median: 3, interquartile range, IQR: 3–4). On the one hand, the data shows an increase in dissatisfaction with the physical performance from 9.7 to 19.3%. In addition, the sensation of very poor or poor condition QoL increased to 9.7% after the increase in cases and deaths due to COVID-19.

The increase in cases and deaths also was associated with moderate safety in daily lives (68.2%, 75 individuals) and a high proportion of participants (93.4%, 103 individuals) experiencing good health in their physical domain. On the other hand, the feeling of completely or mostly having enough money to cover their needs was reduced (from 19.1 to 3.6%). Sleep problems increased with the rising cases of COVID-19 (from 29.1 to 41.9%), and dissatisfaction about the ability to perform daily activities increased from 10 to 12.7%. Most participants had no negative feelings such as sadness, despair, anxiety, or depression (51.8 and 58.1% at the first and second assessments, respectively). Also, we found differences in quality of life according to gender, age group, and type of education (*p* < 0.05).

In the first stage of the study, an overall mean QoL of 46.65 ± 23.2 points were obtained, while for the second stage, it was 35 ± 27.7 points (Table 2). Individuals experienced significantly lower QoL in the face of increased deaths in three of the four QoL domains (*p* = 0.041). The environment dimension was the most affected [51.84 ± 5.81 points for the first stage versus 16.66 ± 5.55 points for the second stage (*p* = 0.001)]. The complete results of the WHOQOL-BREF in the Peruvian jungle population at the two stages of the study are available on Supplementary Table 1.

**TABLE 2:** Global and inter-dimensional quality of life in individuals from the Peruvian jungle during COVID-19.

	Study assessments		
**Quality of life**	**First (April)**	**Second (June)**	***P*-value**
	**Mean ± SD**	**Mean ± SD**	
Physical health	23.19 ± 8.20	16.66 ± 5.78	0.001
Psychological	34.97 ± 7.34	31.72 ± 6.79	0.028
Social relationship	76.61 ± 22.42	75.17 ± 14.75	0.755
Environment	51.84 ± 5.81	16.66 ± 5.55	0.001
Total	46.65 ± 23.18	35.14 ± 27.67	0.041

## 4. Discussion

The results of this study show that following an increase in COVID-19 cases and deaths in the Peruvian jungle, a decrease in quality of life and poorer physical, mental, and environmental health were observed. These significant reductions could lead to an impact on activities of daily living as the pandemic progresses.

To the best of the authors’ knowledge, this is the first study about QoL of Peruvian jungle population. This population has health disparities and complex social, economic, and cultural factors (15, 16, 18) that create a gap in the application of daily community practices, making them more vulnerable. Therefore, the alteration of their environment, customs, and habits by the pandemic can dramatically increase these disparities and community disorganization and lead to unplanned situations. Another strength of the study is that it has used the WHOQOL-BREF (27), an instrument recommended by leading healthcare institutions and widely applied in several countries (30). Unlike previous studies (6), we could analyses changes in QoL in the four dimensions of the WHOQOL-BREF in the indigenous population.

The findings of the present study are supported by the analysis of Melo-Oliveira et al. (5) who showed a worsening of QoL in populations affected by COVID-19. This reduction in QoL has also been associated with an increase in neuropsychological disorders. Nguyen et al. (31) in Vietnam showed that people with COVID-19 reduced their quality of life and increased levels of depression. Similar findings to these were reported by Al Dhaheri et al. (32) in the average population of the Middle East and North Africa and in the study by Shah et al. (33) in Europe and North America. Further investigation is required to link neuropsychological disorders and QoL in the Amazon population.

As our results show, the arrival of the pandemic can reduce the QoL in 3 of 4 dimensions, may be underpinned by pandemic measures, mainly in social distancing (34). Reductions in specific dimensions of QoL were the most striking findings, and thus physical health was severely affected due to movement restrictions imposed in each country (35). Among indigenous peoples, the impact may be greater because intervention programs have limitations in addressing physical and mental health due to management issues (36). The dramatic effects of the pandemic on mental health, pain, and physical activity were recently demonstrated in a multicenter study, with populations mainly in Brazil and Italy showing more changes (37). According to these reports that support our findings, the reduction in QoL in the population of the Peruvian jungle had an impact because of the strict measures against the pandemic implemented during the first wave, mainly affecting the Environment dimension but, interestingly, not the social relationship dimension. It could be explained by the “unusual” quarantine since, although it complies with the health confinement, the quarantine is also a space that favors remote social dialogue (through social networks) (38) or directly with family members (32, 39). Against this backdrop of social distancing and restrictions, digital interconnection is most compelling, allowing communication to be maintained during home isolation (40, 41).

Our findings are supported by several studies of rural populations where COVID-19 has led to a decrease in QoL (10, 11). Residents in these areas also had a low QoL surviving the disease (12), which is consistent with our results, with a plummeting QoL and worsening of the lockdown during the follow-up. The Peruvian Ministry of Health is conducting epidemiological follow-up of cases in indigenous and rural populations (20), but changes in the wellbeing of the inhabitants have not been glimpsed. It is a fact that QoL can vary and be severely reduced with increasing cases and new outbreaks, to the point that recent research on health caregivers in the Peruvian jungle has shown that their poor QoL is associated with symptoms of depression (42). Thus, the trace that the pandemic can leave on indigenous populations can have an impact long afterward if the population is not rehabilitated and organized and decentralized mental health programs are not followed.

Results from other studies have also shown that QoL is significantly reduced due to certain characteristics of group identification (43). These familiar, religious, and national aspects may be determinants of the wellbeing and mental health in rural and peri-urban communities, as there are well-marked differences from urban populations (access to health care facilities, higher economic income, etc.). During the COVID-19 pandemic, individuals with remote access to health care (44), low literacy rates (31), and higher infection rates (45) experienced lower levels of quality of life. These adverse traits related to wellbeing may be present in the Peruvian jungle population analyzed in this study, as this population was heavily beaten by COVID-19 during the first wave (24).

There are limitations to the current study. First, the study was conducted during the global outbreak of COVID-19, and both interviewers and volunteer participants were vulnerable to coronavirus infection. During the interview PPE was used but since the families were in quarantine many did not want to receive visits from the interviewers. This reduced the number of participants. Second, while the sample size was adequate, the health emergency limited the participation of many more participants because many lived in remote, hard-to-reach areas and were not allowed to have contact with researchers.

## 5. Conclusion

Quality of life of indigenous people of jungle of Peru was reduced during periods of increased mortality and incidence by COVID-19. It is important to note that QoL levels in physical, psychological and environmental health were dramatically affected by COVID-19, whereas social relationships did not show significant differences throughout the study. Further studies are needed to explore the causal factors involved in the QoL decrease to target public health interventions to prevent the development of neuropsychological disorders linked to low QoL during the COVID-19 pandemic.

## Data availability statement

The datasets presented in this study can be found in online repositories. The names of the repository/repositories and accession number(s) can be found below: Figshare (doi: 10.6084/m9.figshare.19425941.v1).

## Ethics statement

The studies involving human participants were reviewed and approved by the IRB of the Universidad Norbert Wiener (Document FCE-RRR-COVID-2020.03-01). The patients/participants provided their written informed consent to participate in this study.

## Author contributions

JM-S and CV completed the raw data collection and processing. JM-S, CV, BC, and HC-P performed the data analysis. JM-S, KC-F, and HC-P wrote and edited the manuscript with input from BM-S and BC. All authors had input in writing and finalizing the survey questions, contributed to distributing the survey, and approved the final manuscript.
