# Supplementary material for: COVID-19 may lower quality of life when infections and deaths increase: A longitudinal study in the Peruvian jungle
Source: Front Psychiatry. 2023 Mar 28;14:905377. doi: 10.3389/fpsyt.2023.905377 (PMC10086153; doi:10.3389/fpsyt.2023.905377)
Supplement: Supplementary file 1 [file Table_1.DOCX]

**Suppl 2.** Baseline responses of the WHOQOL-BREF in the first and second stages of the study. Data in n (%)

| **Items** | | | | **First stage** | | | | | | | | | | | | | **Second stage** | | | | | | | | | | | | |  |  |
| --- | --- | --- | --- | --- | --- | --- | --- | --- | --- | --- | --- | --- | --- | --- | --- | --- | --- | --- | --- | --- | --- | --- | --- | --- | --- | --- | --- | --- | --- | --- | --- |
|  |  |  |  | Very poor | | | Poor | | | Neither poor nor good | | Good | | Very good | | Very poor | | | Poor | | Neither poor or good | | Good | | Very good | | |  |  |  |  |
|  |  |  |  |  |  |  |  |  |  |  |  |  |  |  |  |  |  |  |  |  |  |  |  |  |  |  |  |  |  |  |  |
|  |  |  |  |  |  | | |  | | |  | |  | |  | | |  | |  | |  | |  | | |  | | | |  |
| How would you rate your quality of life? | | | | 0 (0) | | 0 (0) | | | 71 (64.5) | | | 25 (22.7) | | 14 (12.7) | | 7 (6.5) | | | 4 (3.2) | | 74 (67.7) | | 18 (16.1) | | | 7 (6.5) | |  |  |  |  |
|  |  |  |  |  |  | | |  | | |  | |  | |  | | |  | |  | |  | |  | | |  | | | |  |
|  | | | | Very dissatisfied | | | Dissatisfied | | | Neither satisfied or dissatisfied | | Satisfied | | Very satisfied | | Very dissatisfied | | | Dissatisfied | | Neither satisfied or dissatisfied | | Satisfied | | | Very satisfied | |  |  |  |  |
|  | | | |  |  |  |  |  |  |  |  |  |  |  |  |  |  |  |  |  |  |  |  |  |  |  |  |  |  |  |  |
| How satisfied are you with your health? | | | | 0 (0) | | | 11 (9.7) | | | 57 (51.6) | | 28 (25.8) | | 14 (12.9) | | 4 (3.6) | | | 18 (16.4) | | 78 (71) | | 3 (2.7) | | | 7 (6.4) | |  |  |  |  |
|  |  |  |  |  |  | | |  | | |  | |  | |  | | |  | |  | |  | |  | | |  | | | |  |
|  | | | | Not all | | | A little | | | A moderate amount | | Very much | | An extreme amount | | Not all | | | A little | | A moderate amount | | Very much | | | An extreme amount | |  |  |  |  |
|  | | | |  |  |  |  |  |  |  |  |  |  |  |  |  |  |  |  |  |  |  |  |  |  |  |  |  |  |  |  |
| To what extent do you feel that physical pain prevents you from doing what you need to do? | | | | 14 (12.7) | | | 36 (32.7) | | | 42 (38.2) | | 11 (10) | | 7 (6.4) | | 21 (19.1) | | | 25 (22.7) | | 50 (45.5) | | 14 (12.7) | | | 0 (0) | |  |  |  |  |
|  |  |  |  |  |  |  |  |  |  |  |  |  |  |  |  |  |  |  |  |  |  |  |  |  |  |  |  |  |  |  |  |
| How much do you need any medical treatment to function in your daily life? | | | | 18 (16.4) | | | 28 (25.5) | | | 50 (45.5) | | 14 (12.7) | | 0 (0) | | 21 (19.1) | | | 28 (25.5) | | 43 (39.1) | | 11 (10) | | | 7 (6.4) | |  |  |  |  |
| How much do you enjoy life? | | | | 0 (0) | | | 0 (0) | | | 39 (35.5) | | 57 (51.8) | | 14 (12.7) | | 0 (0) | | | 4 (3.6) | | 50 (45.5) | | 56 (50.9) | | | 0 (0) | |  |  |  |  |
| To what extent do you feel your life to be meaningful? | | | | 0 (0) | | | 3 (2.7) | | | 53 (48.2) | | 36 (32.7) | | 18 (16.4) | | 0 (0) | | | 4 (3.6) | | 53 (48.2) | | 46 (41.8) | | | 7 (6.4) | |  |  |  |  |
|  | | | |  | | |  | | |  | |  | |  | |  | | |  | |  | |  | | |  | |  |  |  |  |
|  | | | |  | | |  | | |  | |  | |  | |  | | |  | |  | |  | | |  | |  |  |  |  |
|  | | | | Not all | | | A little | | | A moderate amount | | Very much | | Extremely | | Not all | | | A little | | A moderate amount | | Very much | | | Extremely | |  |  |  |  |
|  | | | |  |  |  |  |  |  |  |  |  |  |  |  |  |  |  |  |  |  |  |  |  |  |  |  |  |  |  |  |
| How well are you able to concentrate? | | | | 0 (0) | | | 18 (16.4) | | | 60 (54.5) | | 25 (22.7) | | 7 (6.4) | | 0 (0) | | | 4 (3.6) | | 64 (58.2) | | 39 (35.5) | | | 3 (2.7) | |  |  |  |  |
| How safe do you feel in your daily life? | | | | 0 (0) | | | 7 (6.4) | | | 53 (48.2) | | 43 (39.1) | | 7 (6.4) | | 0 (0) | | | 4 (3.6) | | 75 (68.2) | | 32 (28.2) | | | 0 (0) | |  |  |  |  |
| How healthy is your physical environment? | | | | 4 (3.6) | | | 7 (6.4) | | | 71 (64.5) | | 28 (25.5) | | 0 (0) | | 0 (0) | | | 7 (6.4) | | 60.3 (54.5) | | 39 (35.5) | | | 4 (3.6) | |  |  |  |  |
|  | | | |  | | |  | | |  | |  | |  | |  | | |  | |  | |  | | |  | |  |  |  |  |
|  | | | | Not all | | | A little | | | Moderately | | Mostly | | Completely | | Not all | | | A little | | Moderately | | Mostly | | | Completely | |  |  |  |  |
|  | | | |  |  |  |  |  |  |  |  |  |  |  |  |  |  |  |  |  |  |  |  |  |  |  |  |  |  |  |  |
| Do you have enough energy for everyday life? | | | | 0 (0) | | | 0 (0) | | | 46 (41.8) | | 53 (49.1) | | 10 (9.1) | | 4 (3.6) | | | 21 (19.1) | | 36 (32.7) | | 49 (44.5) | | | 0 (0) | |  |  |  |  |
| Are you able to accept your bodily appearance? | | | | 0 (0) | | | 7 (6.4) | | | 25 (22.7) | | 53 (48.2) | | 25 (22.7) | | 0 (0) | | | 0 (0) | | 46 (41.8) | | 39 (35.5) | | | 25 (22.7) | |  |  |  |  |
| Have you enough money to meet your needs? | | | | 7 (6.4) | | | 18 (16.4) | | | 64 (58.2) | | 18 (16.4) | | 3 (2.7) | | 0 (0) | | | 28 (25.5) | | 78 (71) | | 4 (3.6) | | | 0 (0) | |  |  |  |  |
| How available to you is the information that you need in your day-to-day life? | | | | 3 (2.7) | | | 18 (16.4) | | | 50 (45.5) | | 32 (29.1) | | 7 (6.4) | | 4 (3.6) | | | 0 (0) | | 78 (71) | | 25 (22.7) | | | 3 (2.7) | |  |  |  |  |
| To what extent do you have the opportunity for leisure activities? | | | | 14 (12.7) | | | 25 (22.7) | | | 53 (48.2) | | 18 (16.4) | | 0 (0) | | 0 (0) | | | 46 (41.8) | | 53 (48.2) | | 11 (10) | | | 0 (0) | |  |  |  |  |
|  | | | |  | | |  | | |  | |  | |  | |  | | |  | |  | |  | | |  | |  |  |  |  |
|  | | | | Very poor | | | Poor | | | Neither poor nor good | | Good | | Very good | | Very poor | | | Poor | | Neither poor or good | | Good | | | Very good | |  |  |  |  |
|  | | | |  |  |  |  |  |  |  |  |  |  |  |  |  |  |  |  |  |  |  |  |  |  |  |  |  |  |  |  |
| How well are you able to get around? | | | | 3 (2.7) | | | 11 (10) | | | 43 (38.1) | | 39 (35.5) | | 14 (12.7) | | 18 (16.4) | | | 32 (29.1) | | 36 (32.7) | | 14 (12.7) | | | 10 (9.1) | |  |  |  |  |
|  | | | |  | | |  | | |  | |  | |  | |  | | |  | |  | |  | | |  | |  |  |  |  |
|  | | | | Very dissatisfied | | | Dissatisfied | | | Neither satisfied or dissafitisfied | | Satisfied | | Very satisfied | | Very dissatisfied | | | Dissatisfied | | Neither satisfied or dissafitisfied | | Satisfied | | | Very satisfied | |  |  |  |  |
|  | | | |  |  |  |  |  |  |  |  |  |  |  |  |  |  |  |  |  |  |  |  |  |  |  |  |  |  |  |  |
| How satisfied are you with your sleep? | | | | 0 (0) | | | 32 (29.1) | | | 39 (35.5) | | 21 (19.1) | | 18 (16.4) | | 7 (6.4) | | | 39 (35.5) | | 46 (41.8) | | 18 (16.4) | | | 0 (0) | |  |  |  |  |
| How satisfied are you with your ability to perform your daily living activities? | | | | 0 (0) | | | 11 (10) | | | 64 (58.2) | | 28 (25.5) | | 7 (6.4) | | 0 (0) | | | 14 (12.7) | | 75 (68.2) | | 18 (16.4) | | | 3 (2.7) | |  |  |  |  |
| How satisfied are you with your capacity for work? | | | | 0 (0) | | | 11 (10) | | | 64 (58.2) | | 25 (22.7) | | 10 (9.1) | | 0 (0) | | | 10 (9.1) | | 58 (52.7) | | 35 (31.8) | | | 7 (6.4) | |  |  |  |  |
| How satisfied are you with yourself? | | | | 0 (0) | | | 7 (6.4) | | | 39 (35.5) | | 50 (45.5) | | 14 (12.7) | | 0 (0) | | | 4 (3.6) | | 46 (41.8) | | 46 (41.8) | | | 14 (12.7) | |  |  |  |  |
| How satisfied are you with your personal relationships? | | | | 0 (0) | | | 14 (12.7) | | | 50 (45.5) | | 39 (35.5) | | 7 (6.4) | | 0 (0) | | | 11 (10) | | 64 (58.2) | | 32 (29.1) | | | 3 (2.7) | |  |  |  |  |
| How satisfied are you with your sex life? | | | | 10 (9.1) | | | 14 (12.7) | | | 40 (36.4) | | 28 (25.5) | | 18 (16.4) | | 11 (10) | | | 14 (12.7) | | 67 (60.9) | | 14 (12.7) | | | 4 (3.6) | |  |  |  |  |
| How satisfied are you with the support you get from your friends? | | | | 14 (12.7) | | | 14 (12.7) | | | 50 (45.5) | | 25 (22.7) | | 7 (6.4) | | 8 (7.3) | | | 20 (18.2) | | 61 (55.5) | | 14 (12.7) | | | 7 (6.4) | |  |  |  |  |
| How satisfied are you with the conditions of your living place? | | | | 0 (0) | | | 4 (3.6) | | | 57 (51.8) | | 21 (19.1) | | 28 (25.5) | | 0 (0) | | | 11 (10) | | 50 (45.5) | | 28 (25.5) | | | 21 (19.1) | |  |  |  |  |
| How satisfied are you with your access to health services? | | | | 0 (0) | | | 7 (6.4) | | | 67 (60.9) | | 21 (19.1) | | 15 (13.6) | | 7 (6.4) | | | 7 (6.4) | | 50 (45.5) | | 36 (32.7) | | | 10 (9.1) | |  |  |  |  |
| How satisfied are you with your transport? | | | | 11 (10) | | | 11 (10) | | | 60 (54.8) | | 18 (16.4) | | 10 (9.1) | | 21 (19.1) | | | 32 (29.1) | | 46 (41.8) | | 11 (10) | | | 0 (0) | |  |  |  |  |
|  | | | |  | | |  | | |  | |  | |  | |  | | |  | |  | |  | | |  | |  |  |  |  |
|  | | | | Never | | | Seldom | | | Quite often | | Very often | | Always | | Never | | | Seldom | | Quite often | | Very often | | | Always | |  |  |  |  |
|  | | | |  |  |  |  |  |  |  |  |  |  |  |  |  |  |  |  |  |  |  |  |  |  |  |  |  |  |  |  |
| How often do you have negative feelings such as blue mood, despair, anxiety, depression? | | | | 14 (12.7) | | | 43 (39.1) | | | 46 (41.8) | | 7 (6.4) | | 0 (0) | | 3 (9.7) | | | 15 (48.4) | | 12 (38.7) | | 1 (3.2) | | | 0 (0) | |  |  |  |  |
|  |  |  |  |  |  |  |  |  |  |  |  |  |  |  |  |  |  |  |  |  |  |  |  |  |  |  |  |  |  |  |  |
|  |  |  |  |  |  | | |  | | |  | |  | |  | | |  | |  | |  | |  | | |  | | | |  |
